# Smartphone App–Based and Paper-Based Patient-Reported Outcomes Using a Disease-Specific Questionnaire for Dry Eye Disease: Randomized Crossover Equivalence Study

**DOI:** 10.2196/42638

**Published:** 2023-08-03

**Authors:** Ken Nagino, Yuichi Okumura, Yasutsugu Akasaki, Kenta Fujio, Tianxiang Huang, Jaemyoung Sung, Akie Midorikawa-Inomata, Keiichi Fujimoto, Atsuko Eguchi, Shokirova Hurramhon, Alan Yee, Maria Miura, Mizu Ohno, Kunihiko Hirosawa, Yuki Morooka, Akira Murakami, Hiroyuki Kobayashi, Takenori Inomata

**Affiliations:** 1 Department of Hospital Administration Juntendo University Graduate School of Medicine Tokyo Japan; 2 Department of Ophthalmology Juntendo University Graduate School of Medicine Tokyo Japan; 3 Department of Digital Medicine Juntendo University Graduate School of Medicine Tokyo Japan; 4 AI Incubation Farm Juntendo University Graduate School of Medicine Tokyo Japan

**Keywords:** dry eye syndrome, mobile app, equivalence trial, Ocular Surface Disease Index, patient-reported outcome measures, mobile health, reliability, validity, telemedicine, precision medicine

## Abstract

**Background:**

Using traditional patient-reported outcomes (PROs), such as paper-based questionnaires, is cumbersome in the era of web-based medical consultation and telemedicine. Electronic PROs may reduce the burden on patients if implemented widely. Considering promising reports of DryEyeRhythm, our in-house mHealth smartphone app for investigating dry eye disease (DED) and the electronic and paper-based Ocular Surface Disease Index (OSDI) should be evaluated and compared to determine their equivalency.

**Objective:**

The purpose of this study is to assess the equivalence between smartphone app–based and paper-based questionnaires for DED.

**Methods:**

This prospective, nonblinded, randomized crossover study enrolled 34 participants between April 2022 and June 2022 at a university hospital in Japan. The participants were allocated randomly into 2 groups in a 1:1 ratio. The paper-app group initially responded to the paper-based Japanese version of the OSDI (J-OSDI), followed by the app-based J-OSDI. The app-paper group responded to similar questionnaires but in reverse order. We performed an equivalence test based on minimal clinically important differences to assess the equivalence of the J-OSDI total scores between the 2 platforms (paper-based vs app-based). A 95% CI of the mean difference between the J-OSDI total scores within the ±7.0 range between the 2 platforms indicated equivalence. The internal consistency and agreement of the app-based J-OSDI were assessed with Cronbach α coefficients and intraclass correlation coefficient values.

**Results:**

A total of 33 participants were included in this study. The total scores for the app- and paper-based J-OSDI indicated satisfactory equivalence per our study definition (mean difference 1.8, 95% CI –1.4 to 5.0). Moreover, the app-based J-OSDI total score demonstrated good internal consistency and agreement (Cronbach α=.958; intraclass correlation=0.919; 95% CI 0.842 to 0.959) and was significantly correlated with its paper-based counterpart (Pearson correlation=0.932, *P*<.001).

**Conclusions:**

This study demonstrated the equivalence of PROs between the app- and paper-based J-OSDI. Implementing the app-based J-OSDI in various scenarios, including telehealth, may have implications for the early diagnosis of DED and longitudinal monitoring of PROs.

## Introduction

Dry eye disease (DED) is the most common disease of the ocular surface, with a prevalence ranging from 5% to 50% [1,2]. DED presents in a highly personalized manner with numerous symptoms, including eye dryness, discomfort, decreased visual acuity, and generalized fatigue [3-5]. These symptoms decrease the quality of life and work productivity, and DED management imposes a burden on families and health care infrastructure [6]. However, DED has no cure, and the current standard of care revolves around the post facto management of subjective symptoms and preventive measures to halt disease progression [7]. A large proportion of patients with DED may be undiagnosed and untreated despite the presence of DED symptoms [8]. Hence, early detection and intervention, followed by longitudinal monitoring, are crucial to preventing disease progression [4,9,10].

The current diagnostic standards proposed by 2 leading organizations on DED, namely the Tear Film & Ocular Surface Society and the Asian Dry Eye Society, suggest a holistic assessment of patients’ subjective symptoms and tear film breakup time (TFBUT) for diagnosing DED [6,11]. Subjective symptoms of DED should be assessed using disease-specific questionnaires to quantify the degree and types of symptoms [12]. Questionnaire results do not always correlate with clinical impressions and are frequently affected by individual lifestyle patterns, habits, and the quality of life [12,13]. Therefore, the longitudinal measurement of subjective symptoms in a true-to-life environment to negate the fluctuations of symptom-based questionnaire scores is necessary to accurately evaluate the patients’ condition and the effectiveness of ongoing treatment [8,14,15].

To date, mobile health (mHealth) [16] research and implementation have augmented the capability of telehealth by screening large populations for early diagnosis and long-term monitoring of chronic illnesses via remote analyses of results [17-19]. Moreover, researchers have investigated the merits of mHealth, including electronic patient-reported outcomes (ePROs), in routine assessments [20,21]. ePROs offer insights into the patients’ subjective experiences, particularly their disease symptoms and satisfaction with the treatment outcomes, which are collected through digital questionnaires [13,21-24]. Traditional patient-reported outcomes (PROs), such as paper-based questionnaires, are cumbersome for collecting daily subjective symptoms in telemedicine and web-based practice settings. Conversely, ePRO reports indicate that the remote accessibility and usability of electronic adaptation reduce the burden on patients and that widely implemented ePROs may be relatively well-accepted by patients [21,23,25,26]. However, the ePRO Good Research Task Force by the Professional Society for Health Economics and Outcomes Research cautions that numerous questionnaires are developed as PRO tools under the assumption of an on-site paper-based administration; thus, a thorough comparison of the 2 platforms is warranted to ensure the reliability of substituting ePRO tools for traditional PRO tools [21,23].

In November 2016, we developed and released DryEyeRhythm, an in-house mHealth smartphone app for DED research [4,8,14,15,27-29]. Other apps on DED screening include Optrex, a web-based blink test app released in 2018, and Dry eye or not? a smartphone app released in Thailand in 2019 [30,31]. Both DryEyeRhythm and Dry eye or not? collect ePROs by using an electronic version of the Ocular Surface Disease Index (OSDI), which helps clinicians assess subjective symptoms of DED on a standardized scale [32]. So far, research studies have facilitated overcoming the challenges of traditional medicine in DED, such as the early recognition of undiagnosed patients with DED and an unintrusive longitudinal analysis of subjective symptoms to generate data in daily life [8,15,33,34]. However, the Professional Society for Health Economics and Outcomes Research recommends a comprehensive evaluation and comparison of electronic- and paper-based OSDI to assess their equivalency.

Therefore, in this study, we aimed to compare the characteristics of the app- and paper-based OSDI and assess the equivalency and validity of the app-based OSDI as an appropriate substitute for the traditional OSDI.

## Methods

### Study Design and Participants

This prospective, nonblinded, randomized crossover study was conducted at the Department of Ophthalmology at Juntendo University Hospital, Tokyo, Japan. Patients aged ≥20 years were recruited between April 20, 2022, and June 8, 2022. Patients with a history of eyelid disorders, ptosis, mental disease, Parkinson disease, or any other disease affecting blinking were excluded. Furthermore, we excluded patients with missing data from the analysis.

### Ethics Approval

Written informed consent was obtained from all participants. This study was approved by the Independent Ethics Committee of Juntendo University Faculty of Medicine (E21-0324-H02) and was conducted in accordance with the ethical standards laid down in an appropriate version of the Declaration of Helsinki (as revised in Brazil, 2013). All the involved parties attempted to protect the personal information and privacy of the participants. Data related to the participants were anonymized, and research data were stored in locked cabinets with access strictly controlled by the research staff. The participants were not compensated for participating in this study.

### DryEyeRhythm Smartphone App

**Figure 1:**
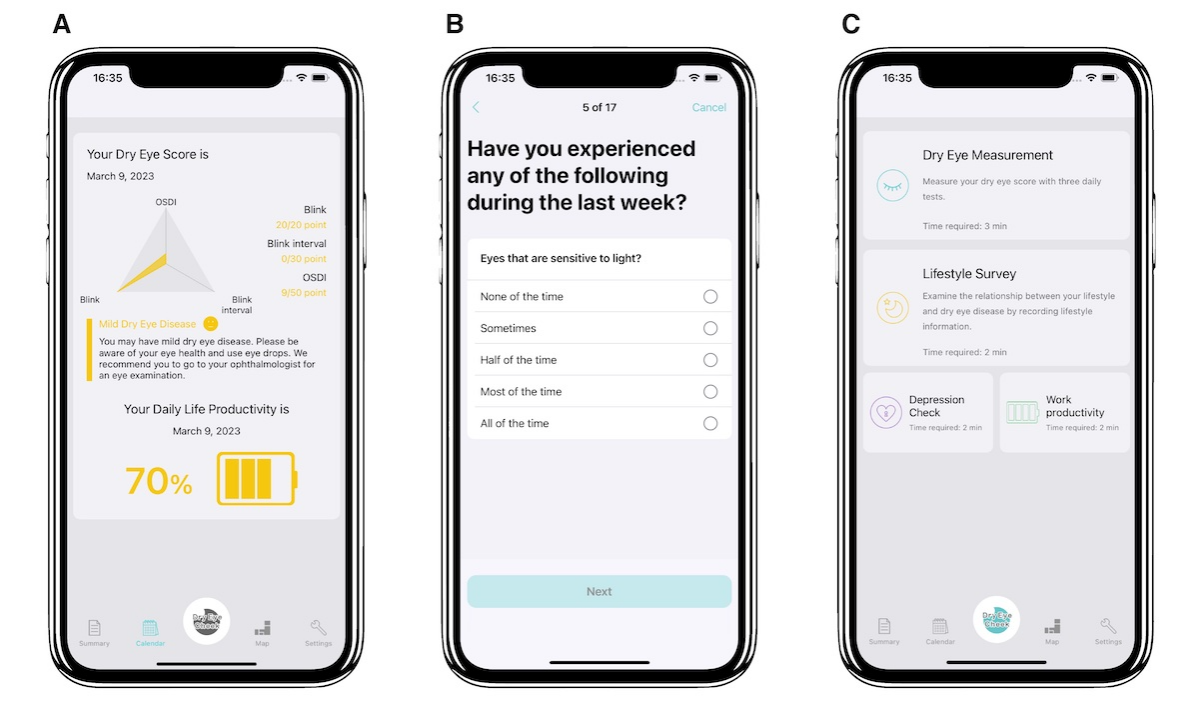
Screenshots of the DryEyeRhythm app. (A) Screenshot of the DryEyeRhythm test results. (B) Screenshot of the DryEyeRhythm app-based J-OSDI. (C) Screenshot of the DryEyeRhythm measuring menu. J-OSDI: Japanese version of the Ocular Surface Disease Index.

The DryEyeRhythm app was developed using the open-source framework ResearchKit (Apple Inc; Figure 1) [14]. This app was released in November 2016 and September 2020 for iOS and Android, respectively, under a consignment contract with the Juntendo University Graduate School of Medicine and InnoJin Inc. It is freely available on Apple’s App Store and Google Play. The DryEyeRhythm app collects data regarding user demographics, medical history, lifestyle questionnaires, daily subjective symptoms, the Japanese version of the OSDI (J-OSDI) questionnaire (Figure 1), blink sensing, the Zung Self-Rating Depression questionnaires for depression, and the Work Productivity and Activity Impairment Questionnaire for work productivity (Figure 1) [3,4,8,14,15,35]. In this study, we assessed only the J-OSDI collected through the app for its equivalence, reliability, and validity compared with the paper-based J-OSDI, and we did not use data on the remaining functions.

### Study Procedures

**Figure 2:**
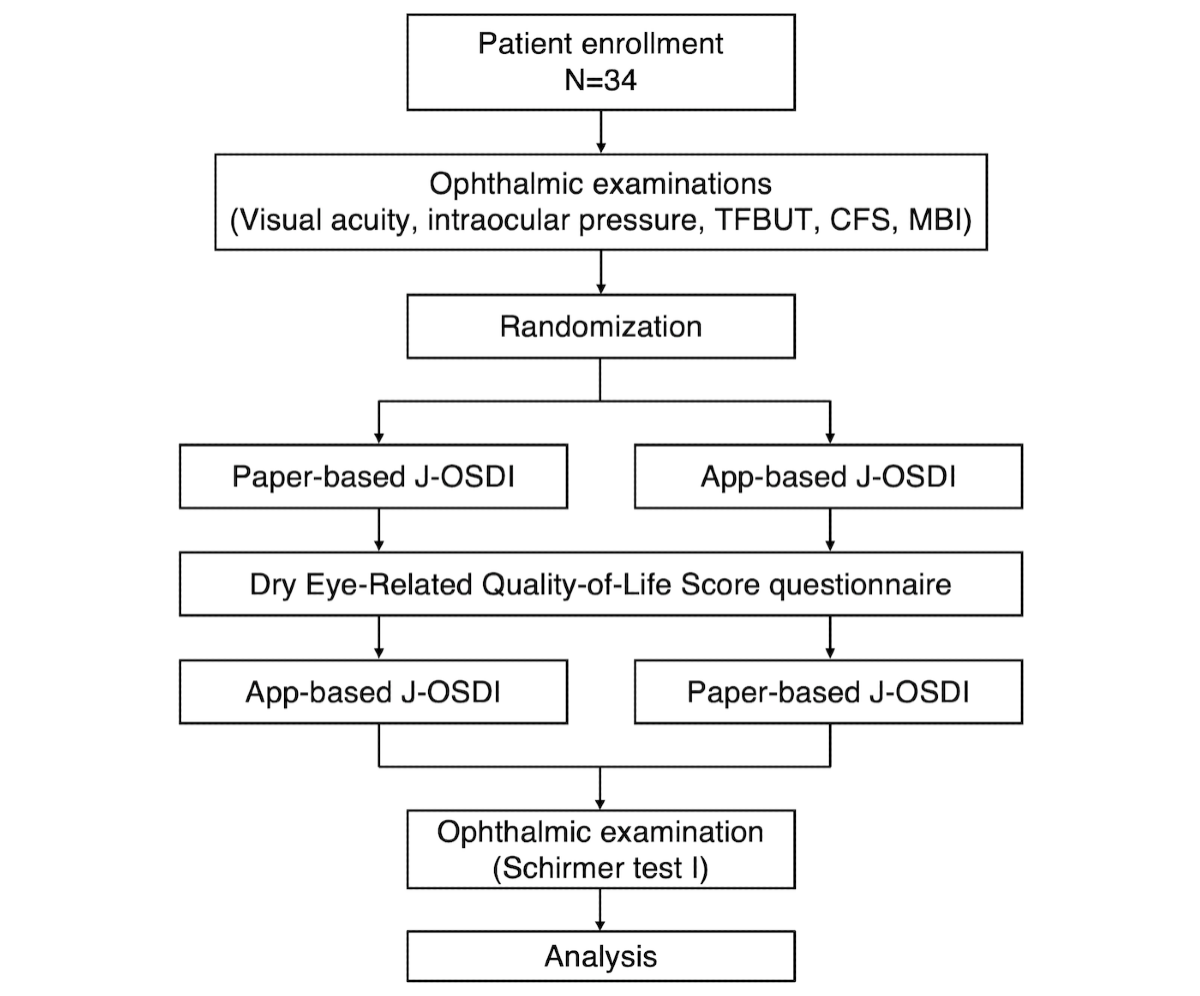
Study schema. CFS: corneal fluorescein staining; J-OSDI: Japanese version of the Ocular Surface Disease Index; MBI: maximum blink interval; TFBUT: tear film breakup time.

Figure 2 depicts the study schema. All participants underwent visual acuity measurements, intraocular pressure measurements, and other DED examinations, including TFBUT, corneal fluorescein staining (CFS), and maximum blink interval (MBI). Subsequently, the participants were allocated randomly into (1) the paper-app group and (2) the app-paper group in a 1:1 ratio. Patients in the paper-app group initially responded to the paper-based J-OSDI, followed by the app-based J-OSDI through DryEyeRhythm. Those in the app-paper group initially responded to the app-based J-OSDI through DryEyeRhythm, followed by the paper-based J-OSDI. Each participant was requested to complete both versions of the J-OSDI. All participants responded to the app-based J-OSDI questionnaire on their own by tapping on the screen of a smartphone with preinstalled DryEyeRhythm (Figure 1). They responded to the Dry Eye-Related Quality-of-Life Score (DEQS) questionnaire before responding to the second round of J-OSDI (app-based J-OSDI for those who began with the paper-based J-OSDI and vice versa).

### Assessment of Subjective DED Symptoms

Subjective DED symptoms were assessed using the J-OSDI and DEQS questionnaires. The J-OSDI is used for assessing subjective DED symptoms; it is a 12-item instrument with the following 3 subscales: ocular symptoms, vision-related functions, and environmental triggers [3]. The J-OSDI questionnaire has been validated in Japan [3]. It records the frequency of each symptom on a 5-point scale from “all of the time” (a score of 4) to “none of the time” (a score of 0). The patients selected “not applicable” if the questions 6 to 12 were irrelevant. The J-OSDI total score and each subscale score ranged from 0 to 100 points and were separately reported [3].

The DEQS questionnaire was administered to the participants to assess DED symptom severity and the multifaceted effects of DED on daily life [36]. The DEQS is a subjective measurement of DED symptoms; 0 and 100 points indicate the best (no symptoms) and worst (maximum symptoms) scores, respectively.

### Clinical Assessment of DED

We performed DED examinations using the TFBUT, CFS, MBI measurements, the Schirmer test I, and Meibomian gland dysfunction assessment [4].

TFBUT was measured using fluorescein sodium staining (fluorescence ocular examination test paper; Ayumi Pharmaceutical Co) [11]. The mean values of the 3 measurements were used.

CFS was evaluated according to the van Bijsterveld grading system [37], which divides the ocular surface into the 3 following zones: the nasal bulbar conjunctiva, the temporal bulbar conjunctiva, and the cornea. Each zone was evaluated on a scale ranging from 0 to 3, with 0 indicating no staining and 3 indicating confluent staining; the maximum score was 9.

MBI was defined as the duration for which the participants could keep their eyes open before blinking [38]. We measured MBI twice using a stopwatch under a light microscope; MBI was recorded at 30 seconds if it exceeded 30 seconds.

We performed Schirmer test I without topical anesthesia after completing other examinations. Schirmer test strips (Ayumi Pharmaceutical Co) were placed on the outer third of the temporal lower conjunctival fornix for 5 minutes. These strips were removed, and the length (in mm) of the dampened filter paper was recorded [39].

Meibomian gland function was assessed by applying digital pressure onto the lower central eyelid in conjunction with slit-lamp microscopy, according to the standard method [40].

### DED Diagnosis

DED and non-DED were diagnosed using the 2016 Asian Dry Eye Society and Tear Film & Ocular Surface Society Dry Eye Workshop II diagnostic criteria [6,11]. The diagnosis was based on the following 2 findings: positive subjective symptoms (paper-based J-OSDI total score ≥13) and decreased TFBUT (≤5.0 seconds).

### Randomization

The participants were randomized by simple random sampling using the lottery method [41]. The total sample size was determined to be 34, as described in the Statistical Analyses section. To assign participants to their respective groups, shuffled cards numbered from 1 to 34 were drawn randomly from an opaque envelope. Those who drew odd and even numbers were assigned to the paper-app and app-paper groups, respectively [42].

### Statistical Analyses

The sample size was predetermined based on the methodology presented in the Professional Society for Health Economics and Outcomes Research ePRO Good Research Practices Task Force report [21]. The sample size required for crossover design comparisons of means from 2 different PRO administration modes is calculated by multiplying the total sample size required for a parallel group design by a factor of (1–*r*)/2, where *r* is an estimate of the expected correlation between the 2 modes of administration [21]. With a power of 80%, a significance level of 5%, a minimal clinically important difference (MCID) of 7.0 points for the J-OSDI total score [43], an SD of 20.0 for the paper-based J-OSDI score [4], and a correlation coefficient of 0.89 between the paper- and app-based J-OSDI [4], the required sample size was calculated as 30 (15 cases per group [21]). For 34 cases (17 in each group), we considered 10% dropouts because of missing data or the withdrawal of consent.

The equivalence margin was defined as ±7.0 points from the MCID of the J-OSDI total score [43]. A 95% CI of the mean difference between the J-OSDI total scores of the app- and paper-based J-OSDI within the ±7.0 range denoted equivalence [44,45].

We assessed the internal consistency of the app-based J-OSDI using the Cronbach α coefficient [4]. Cronbach α>.70 was considered acceptable [46]. The intraclass correlation coefficient (ICC) was used to evaluate the agreement of the J-OSDI total score and subscale scores between the app- and paper-based J-OSDI. An ICC value ≥0.70 was considered acceptable [47]. To assess the agreement and correlation between the app- and paper-based J-OSDI, we performed Bland-Altman analysis and Pearson correlation coefficient estimation.

To compare the characteristics of the participants between the app-paper and paper-app groups, we conducted an unpaired 2-tailed *t* test and a chi-squared test for continuous and categorical variables, respectively. All analyses were performed using the STATA software package (version 17.0; StataCorp). Statistical significance was set at *P*<.05.

## Results

### Participant Characteristics

We enrolled 34 patients; we excluded 1 patient because of missing data caused by a poor internet connection. Table 1 summarizes the characteristics of the 33 participants. The mean age was 63.6 years, and 32 (97%) participants were female. The number of participants with DED in the paper-app and app-paper groups was 8 (50%) and 10 (59%), respectively. We observed no statistically significant differences in the demographic characteristics or results of ophthalmological examinations between the groups.

**Table 1:** Participant characteristics.

	Total (n=33)	Paper-app group (n=16)	App-paper group (n=17)	*P* value^a^
Age (years), mean (SD)	63.6 (12.9)	66.2 (10.7)	61.3 (14.6)	.29
Female, n (%)	32 (97)	16 (100)	16 (94)	.32
DED^b^, n (%)	18 (55)	8 (50)	10 (58)	.61
BCVA^c^ (LogMAR), mean (SD)	–0.043 (0.073)	–0.044 (0.072)	–0.043 (0.076)	.98
IOP^d^ (mm Hg), mean (SD)	13.8 (3.8)	13.4 (4.6)	14.3 (2.8)	.52
Paper-based J-OSDI^e^ total score (0-100), mean (SD)	33.6 (24.2)	29.6 (24.2)	37.3 (24.3)	.37
App-based J-OSDI total score (0-100), mean (SD)	31.8 (20.4)	27.1 (20.1)	36.3 (20.1)	.20
DEQS^f^ summary score, (0-100), mean (SD)	34.3 (21.3)	29.9 (20.5)	38.5 (21.7)	.25
TFBUT^g^ (s), mean (SD)	3.0 (1.7)	3.4 (2.0)	2.7 (1.4)	.23
CFS^h^ (0-9), mean (SD)	3.5 (2.8)	3.4 (2.5)	3.5 (3.0)	.88
MBI^i^ (s), mean (SD)	8.3 (4.9)	8.5 (4.3)	8.1 (5.5)	.81
Schirmer test I (mm), mean (SD)	7.4 (8.4)	9.3 (10.9)	5.6 (4.5)	.21
MGD^j^, n (%)	0 (0)	0 (0)	0 (0)	—^k^

^a^*P* values were estimated using the unpaired 2-tailed *t* test for continuous variables and chi-squared test for categorical variables.

^b^DED: dry eye disease.

^c^BCVA: best-corrected visual acuity.

^d^IOP: intraocular pressure.

^e^J-OSDI: Japanese version of the Ocular Surface Disease Index.

^f^DEQS: Dry Eye-Related Quality-of-Life Score.

^g^TFBUT: tear film breakup time.

^h^CFS: corneal fluorescein staining.

^i^MBI: maximum blink interval.

^j^MGD: meibomian gland dysfunction.

^k^Not available.

### Equivalence Test of App- and Paper-Based J-OSDI Based on the MCID

Table 2 summarizes the J-OSDI scores for each question and the mean differences of the scores between the paper-app and app-paper groups. The mean difference in the J-OSDI total score between the 2 groups was 1.8 (95% CI –1.4 to 5.0). Results of the equivalence test based on an MCID of 7.0 demonstrated that the app- and paper-based J-OSDI total scores were equivalent.

**Table 2:** Comparison of the J-OSDI^a^ scores on each question between the paper-app and app-paper groups.

		Paper-app group		App-paper group		Paper- vs app-based J-OSDI, mean difference (95% CI)
		Paper-based J-OSDI, mean (SD)	App-based J-OSDI, mean (SD)	App-based J-OSDI, mean (SD)	Paper-based J-OSDI, mean (SD)	
J-OSDI total score (0-100)		29.6 (24.2)	27.1 (20.1)	36.3 (20.1)	37.3 (24.3)	1.8 (–1.4 to 5.0)
**Ocular symptoms (0-100)**		26.6 (20.9)	20.6 (14.1)	35.0 (19.4)	38.2 (26.5)	4.6 (–0.6 to 9.7)
	1. Eyes that are sensitive to light? (0-4)	1.4 (1.1)	1.1 (1.1)	1.6 (1.3)	1.7 (1.4)	0.2 (–1.2 to 0.6)
	2. Eyes that feel gritty? (0-4)	0.9 (1.2)	0.7 (0.9)	1.8 (1.2)	1.6 (1.3)	0.0 (–0.2 to 0.3)
	3. Painful or sore eyes? (0-4)	0.6 (1.0)	0.6 (0.9)	1.2 (1.2)	1.3 (1.4)	0.1 (–0.3 to 0.4)
	4. Blurred vision? (0-4)	1.2 (1.0)	0.9 (0.8)	1.2 (0.9)	1.4 (1.0)	0.3 (0.0 to 0.4)
	5. Poor vision? (0-4)	1.3 (1.0)	0.9 (0.9)	1.2 (0.9)	1.6 (1.2)	0.4 (0.1 to 0.7)
**Vision-related function (0-100)**		23.8 (26.1)	22.9 (24.7)	29.0 (17.2)	24.9 (21.4)	–1.6 (–7.8 to 4.6)
	6. Reading? (0-4)	1.2 (1.3)	1.0 (1.2)	1.4 (1.2)	1.3 (1.2)	0.1 (–0.4 to 0.4)
	7. Driving at night? (0-4)	1.2 (1.6)	1.5 (1.6)	0.9 (1.1)	0.8 (1.2)	-0.2 (-0.5 to 0.3)
	8. Working with a computer or bank machine? (0-4)	0.9 (1.0)	0.9 (1.1)	1.3 (1.3)	0.9 (1.1)	–0.2 (–0.6 to 0.1)
	9. Watching television? (0-4)	0.9 (1.1)	0.8 (1.0)	0.8 (0.7)	1.1 (1.1)	0.2 (–0.1 to 0.3)
**Environmental triggers (0-100)**		42.7 (37.1)	43.2 (33.5)	45.8 (33.6)	49.3 (37.0)	1.5 (–2.6 to 5.7)
	10. Windy conditions? (0-4)	1.7 (1.7)	1.8 (1.6)	1.7 (1.6)	2.0 (1.7)	0.1 (–0.2 to 0.3)
	11. Places or areas with low humidity (very dry)? (0-4)	1.9 (1.6)	2.0 (1.5)	2.2 (1.3)	2.2 (1.5)	–0.1 (–0.2 to 0.2)
	12. Areas that are air conditioned? (0-4)	1.7 (1.5)	1.6 (1.4)	1.8 (1.3)	1.8 (1.4)	0.1 (–0.2 to 0.4)

^a^J-OSDI: Japanese version of the Ocular Surface Disease Index.

### Internal Consistency and Agreement of the App-Based J-OSDI

Table S1 in Multimedia Appendix 1 summarizes the internal consistency and agreement of the app-based J-OSDI total score and subscale scores with the Cronbach α coefficients and ICC values. The J-OSDI total score (.958), ocular symptoms (.873), vision-related function (.819), and environmental triggers subscales (.971) had Cronbach α coefficients >0.7, which indicated acceptable internal consistency. The ICC values for the J-OSDI total score, ocular symptoms subscale, vision-related function subscale, and environmental triggers subscale were 0.919 (95% CI 0.842-0.959), 0.775 (95% CI 0.592-0.882), 0.693 (95% CI 0.463-0.836), and 0.944 (95% CI 0.890-0.972), respectively. All ICCs, except for the vision-related function subscale, were >0.7.

### Correlation and Agreement Between the App- and Paper-Based J-OSDI

**Figure 3:**
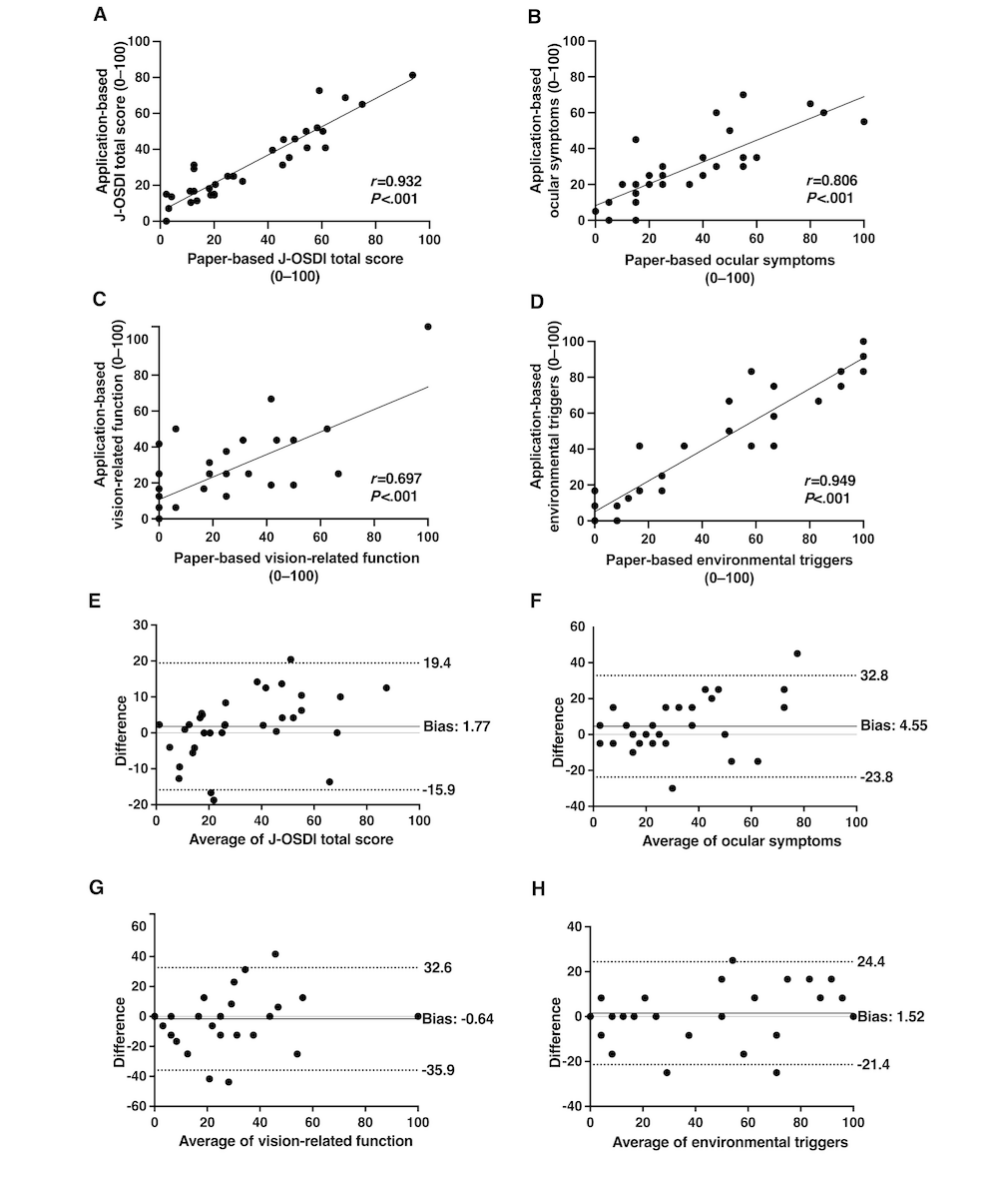
Correlation and agreement between the app- and paper-based J-OSDI. The x-axes of the Bland-Altman plots indicate the average scores of the 2 questionnaires; the y-axes indicate the differences between the scores (paper- and app-based J-OSDI). A, B, C, and D show the Pearson correlation coefficient between the app- and paper-based J-OSDI for total score, ocular symptoms subscale, vision-related function subscale, and the environmental triggers subscale, respectively. E, F, G, and H show Bland-Altman analyses for agreement between the paper- and app-based J-OSDI for total score, the ocular symptoms subscale, the vision-related function subscale, and the environmental triggers subscale, respectively. J-OSDI: Japanese version of the Ocular Surface Disease Index.

Figure 3 depicts the correlation and agreement between the paper- and app-based J-OSDI. We observed a significant positive correlation between the paper- and app-based J-OSDI in the J-OSDI total score (*r*=0.932, *P*<.001) and in each subscale (ocular symptoms: *r*=0.806, *P*<.001; vision-related function: *r*=0.697, *P*<.001; and environmental triggers: *r*=0.949, *P*<.001). The Bland-Altman analysis for agreement between the paper- and app-based J-OSDIs demonstrated differences (biases) of 1.77 (95% limits of agreement [LOA] –15.9 to 19.4) for the J-OSDI total score (Figure 3) and 4.55 (95% LOA –23.8 to 32.8), –0.64 (95% LOA –35.9 to 32.6), and 1.52 (95% LOA –21.4 to 24.4) for the ocular symptoms, vision-related function, and environmental triggers subscales, respectively.

## Discussion

### Principal Findings

In this study, we compared the performance of paper- and app-based J-OSDI through data collected from a DED mHealth app (DryEyeRhythm) to evaluate their equivalency for subjective symptom questionnaires. The app-based J-OSDI total score was comparable to its paper-based counterpart. The recent COVID-19 pandemic limited health care visits worldwide; therefore, efforts to improve telehealth and produce noncontact medical devices are escalating [48]. Evaluating subjective symptoms through an app-based questionnaire may facilitate the implementation of telehealth in DED diagnosis, thus reducing the reliance on in-patient consultations for DED diagnosis and making follow-up simpler for susceptible populations. As a novel mHealth app in Japan, DryEyeRhythm may offer the advantages of early DED diagnosis and effective disease management.

The app- and paper-based versions of the J-OSDI yielded comparable results, suggesting satisfactory performance of the app-based OSDI as a substitute for the counterpart platform. Subjective DED symptoms can be highly variable and nuanced [8,15], and the verbal inquisition of patient experiences can yield unreliable results lacking standardization and quantification. Hence, researchers recommend vetted, disease-specific questionnaires for assessing subjective symptoms as part of the diagnostic process [12]. The J-OSDI total score is often used in diagnosing DED according to the current DED diagnosis guidelines [3,49]. The mean difference in the J-OSDI total score between the 2 platforms was 1.8. A similar study that compared a web-based Chinese version of the OSDI to its paper-based counterpart reported a mean difference of 0.24, lower than our results [50]. Notably, the mean age of the participants in the Chinese study was relatively lower (27.9 years), and the web-based OSDI could display all 12 questions on a single page. In our study, the mean age was higher (63.6 years), which may imply that our participants were less familiar with modern devices. In addition, the user interface in DryEyeRhythm limited the visibility of the questionnaire because of the screen size, and questions were delivered one-by-one sequentially while responding. The difference in interaction with paper-based and digital platforms may result in a discrepancy in the data collected between traditional PROs and ePROs [21], which may lead to relatively higher mean differences between the app- and paper-based J-OSDI results. The J-OSDI total score increases in 2.5 increments each time a participant responds to an item in the questionnaire [3]. The mean difference of 1.8 between the 2 platforms was lower than a single-point change in the OSDI questionnaire. Although the mean difference is higher than the previously reported OSDI discrepancy between its app and paper versions, the clinical significance of the score gap is considered minimal in practice [3]. Thus, the app-based and paper-based J-OSDI total scores yielded comparable results. This finding supports the candidacy of the app-based J-OSDI as a suitable substitute for the paper-based J-OSDI for evaluating DED subjective symptoms.

The app-based J-OSDI demonstrated satisfactory internal consistency, and a comparison of the 2 platforms of J-OSDI indicated high agreement and positive correlation. Notably, the Cronbach α obtained in this study was higher than that obtained in a previous study on the reliability of the paper-based J-OSDI [3]. Similar results were observed for the ICC, except for the vision-related function subscale of the J-OSDI categories [3]. Numerous studies on OSDI adaptations into various languages besides Japanese did not demonstrate a deficit in internal consistency and agreement in the vision-related function subscale [51,52]. Interestingly, while the Cronbach α was lower in the vision-related function subscale compared with other categories in the Japanese study, the exclusion of item 7 (nighttime driving difficulty) noticeably improved the internal consistency [3]. We had a similar observation, in which the ICC increased beyond the 0.7 threshold for an acceptable range of agreement after eliminating item 7 from the analysis. This observation could be attributed to the study demographics consisting of older female participants who tend to drive less frequently during the night and the highly urbanized research site, which allowed easy access to public transportation [3,53]. The resultant Bland-Altman plot provided no evidence for systematic error that could challenge the agreement between the 2 platforms. Therefore, ePRO data collected through app-based J-OSDI was comparable to its traditional paper-based PRO counterpart, signifying the potential of app-based OSDI in mHealth for identifying undiagnosed patients with DED and obtaining subjective symptoms within the users’ daily lives in a nonintrusive manner.

### Limitations

This study had several limitations. First, it may have a selection bias caused by the single-center design at a university hospital in Tokyo, Japan. In addition, most participants were older women. This bias in the target population may have affected the results of J-OSDI item 7. Conversely, the bias observed in the participant group toward older women might have minimally affected our remaining results because DED has a higher prevalence in older women [1]. Furthermore, the older population may not be skilled in using modern digital devices [54]. However, with growing resources and the normalization of smartphone use in daily life, older adults are expected to become more skilled in the use of digital devices in the near future [15,48]. Second, a carryover effect may have influenced our results because the participants may not have had a sufficient washout period before transitioning between the 2 platforms [55]. The interval between responding to the app-based J-OSDI, DEQS, and paper-based J-OSDI questionnaires—or in reverse order—was approximately 10 minutes. However, the participants responded to a non-OSDI questionnaire (DEQS) during the interim period, which could have reduced the carryover effect. Third, participant factors, including socioeconomic status, educational level, and cultural background, were not collected in this study, and researchers should attempt to collect and analyze their effects on outcomes in the future. Fourth, we did not compare the efficiency, effectiveness, or usability of the paper- and app-based J-OSDI. Future studies should demonstrate the response time to the J-OSDI questionnaire, its effectiveness in treating DED, and its usability to establish the performance of the app-based J-OSDI. Fifth, the equivalence assessment between the app- and paper-based J-OSDI was conducted with a relatively small participant pool. This is because a study with a crossover design can often be effectively conducted even with a smaller sample size. However, the validity and reliability of the app-based J-OSDI questionnaire should be evaluated comprehensively with a large sample size, and future researchers should attempt to validate these results with more participants.

### Conclusions

In conclusion, the app-based J-OSDI and the paper-based J-OSDI were comparable in obtaining data on subjective DED symptoms. Implementing the app-based J-OSDI as a tool for the mHealth management of DED may have implications for the early diagnosis of DED and longitudinal PRO monitoring through an unintrusive collection of DED-related data in daily life.

## Appendix

Multimedia Appendix 1 Supplementary Table 1. Comparison of the reliability of the app- and paper-based versions of the J-OSDI.

Multimedia Appendix 2 CONSORT-eHEALTH checklist (V 1.6.1).

## Abbreviations

CFS: corneal fluorescein staining
DED: dry eye disease
DEQS: Dry Eye-Related Quality-of-Life Score
ePRO: electronic patient-reported outcome
ICC: intraclass correlation coefficient
J-OSDI: Japanese Ocular Surface Disease Index
LOA: limits of agreement
MBI: maximum blink interval
MCID: minimal clinically important difference
mHealth: mobile health
OSDI: Ocular Surface Disease Index
PRO: patient-reported outcome
TFBUT: tear film breakup time

## Data Availability

All data generated or analyzed during this study are included in this published article and its supplementary information files.
