# Supplementary material for: Smartphone App–Based and Paper-Based Patient-Reported Outcomes Using a Disease-Specific Questionnaire for Dry Eye Disease: Randomized Crossover Equivalence Study
Source: J Med Internet Res. 2023 Aug 3;25:e42638. doi: 10.2196/42638 (PMC10436120; doi:10.2196/42638)
Supplement: Multimedia Appendix 1 [file jmir_v25i1e42638_app1.pdf]

Supplementary Data

SUPPLEMENTARY TABLE

Supplementary Table 1. Reliability between the app- and paper-based J-OSDI

	**Number of items**	**Cronbach alpha *n*=33**	**ICC (95% CI) *n*=33**
App-based J-OSDI total score	12	0.958	0.919 (0.842–0.959)
Ocular symptoms	5	0.873	0.775 (0.592–0.882)
Vision-related function	4	0.819	0.693 (0.463–0.836)
Environmental triggers	3	0.971	0.944 (0.890–0.972)

Abbreviations: J-OSDI: Japanese version of the Ocular Surface Disease Index; ICC: intraclass correlation coefficient; and CI: confidence interval.
